# Robotic Technologies for High-Throughput Plant Phenotyping: Contemporary Reviews and Future Perspectives

**DOI:** 10.3389/fpls.2021.611940

**Published:** 2021-06-25

**Authors:** Abbas Atefi, Yufeng Ge, Santosh Pitla, James Schnable

**Affiliations:** ^1^Department of Biological Systems Engineering, University of Nebraska–Lincoln, Lincoln, NE, United States; ^2^Department of Agronomy and Horticulture, University of Nebraska–Lincoln, Lincoln, NE, United States

**Keywords:** autonomous robotic technology, agricultural robotics, phenotyping robot, high-throughput plant phenotyping, computer vision

## Abstract

Phenotyping plants is an essential component of any effort to develop new crop varieties. As plant breeders seek to increase crop productivity and produce more food for the future, the amount of phenotype information they require will also increase. Traditional plant phenotyping relying on manual measurement is laborious, time-consuming, error-prone, and costly. Plant phenotyping robots have emerged as a high-throughput technology to measure morphological, chemical and physiological properties of large number of plants. Several robotic systems have been developed to fulfill different phenotyping missions. In particular, robotic phenotyping has the potential to enable efficient monitoring of changes in plant traits over time in both controlled environments and in the field. The operation of these robots can be challenging as a result of the dynamic nature of plants and the agricultural environments. Here we discuss developments in phenotyping robots, and the challenges which have been overcome and others which remain outstanding. In addition, some perspective applications of the phenotyping robots are also presented. We optimistically anticipate that autonomous and robotic systems will make great leaps forward in the next 10 years to advance the plant phenotyping research into a new era.

## Introduction: Robotic Technology Is Vital for High-Throughput Plant Phenotyping

Agriculture must produce enough food, feed, fiber, fuel, and fine chemicals in next century to meet the needs of a growing population worldwide. Agriculture will face multiple challenges to satisfy these growing human needs while at the same time dealing with the climate change, increased risk for drought and high temperatures, heavy rains, and degradation of arable land and depleting water resources. Plant breeders seek to address these challenges by developing high yielding and stress-tolerance crop varieties adapted to future climate conditions and resistant to new pests and diseases (Fischer, 2009; Furbank and Tester, 2011; Rahaman et al., 2015). However, the rate of crop productivity needs to be increased to meet projected future demands. Advances in DNA sequencing and genotyping technologies have relieved a major bottleneck in both marker assisted selection and genomic prediction assisted plant breeding, the determination of genetic information for newly developed plant varieties. Dense genetic marker information can aid in the efficiency and speed of the breeding process (Wang et al., 2018; Happ et al., 2019; Moeinizade et al., 2019). However, large and high quality plant phenotypic datasets are also necessary to dissect the genetic basis of quantitative traits which are related to growth, yield and adaptation to stresses (McMullen et al., 2009; Jannink et al., 2010; Phillips, 2010; Fahlgren et al., 2015; Tripodi et al., 2018; Chawade et al., 2019).

Plant phenotyping is the quantitative and qualitative assessment of the traits of a given plant or plant variety in a given environment. These traits include the biochemistry, physiology, morphology, structure, and performance of the plants at various organizational scales. Plant traits are determined by both genetic and environmental factors as well as non-additive interactions between the two. In addition, variation in one phenotypic trait (e.g., leaf characteristics) can result in variation in other plant traits (e.g., plant biomass or yield). Therefore, phenotyping large numbers of plant varieties for multiple traits across multiple environments is an essential task for plant breeders as they work to select desirable genotypes and identify genetic variants which provide optimal performance in diverse and changing target environments (Granier and Tardieu, 2009; Dhondt et al., 2013; Li et al., 2014; Foix et al., 2015; Walter et al., 2015; Costa et al., 2019; Pieruschka and Schurr, 2019).

Traditionally plant traits are quantified using manual and destructive sampling methods. These methods are usually labor-intensive, time-consuming, and costly. In addition, manual sampling and analysis protocols generally involve many steps requiring human intervention, with each step increasing the chances of introducing mistakes. Often the plant and its organ is cut at fixed time points or at particular phenological stages in order to measure its phenotypic traits. This method destroys or damages the plant at one time point, disallowing the temporal examination of the traits for individual plants during the growing season. For example, yield measurement (such as plant biomass and grain weight) is invasive and more labor intensive compare to the measurement of plant height and leaf chlorophyll content (measured by a handheld sensor). As a result of the labor and resource intensive nature of plant phenotyping, many plant breeders rely solely on a single measurement most critical to their efforts: yield. However, yield is considered as one of the most weakly inherited phenotypes in crop breeding (Richards et al., 2010; Furbank and Tester, 2011). The measurement of other traits in addition to yield can increase the accuracy with which yield can be predicted across diverse environments. Enabling high-throughput and non-destructive measurements of plant traits from large numbers of plants in multiple environments would therefore lead to increases in breeding efficiency (McMullen et al., 2009; Andrade-Sanchez et al., 2013; Fahlgren et al., 2015; Foix et al., 2018; Vijayarangan et al., 2018; Ge et al., 2019; Hassanijalilian et al., 2020a).

In recent years, high-throughput systems and workflows have been developed to monitor and measure large populations of plants rapidly in both greenhouse and field environments. These systems combine modern sensing and imaging modalities with the sensor deployment technologies (including conveyor belts, ground and aerial vehicles, and field gantries) to enable fast measurement and wide area coverage (Busemeyer et al., 2013; Ge et al., 2016; Virlet et al., 2017; Hassan et al., 2019). Although not fully autonomous, these systems represent the state of the art in modern plant phenotyping with several advantages over the traditional, manually collected phenotypic traits.

Robotic systems have been playing a more significant role in modern agriculture and considered as an integral part of precision agriculture or digital farming (Wolfert et al., 2017; Chlingaryan et al., 2018; Zhang et al., 2019; Hassanijalilian et al., 2020b; Jin et al., 2020; Pandey et al., 2021). The robots are fully autonomous and do not need experienced operators to accomplish farming tasks. This is the biggest advantage of the robots compared to tractor-based systems (White et al., 2012). Autonomous robots have taken over a wide range of farming operations including harvesting [Arad et al., 2020 (sweet pepper); Hemming et al., 2014 (sweet pepper); Lili et al., 2017 (tomato); van Henten et al., 2002 (cucumber); Hayashi et al., 2010; Xiong et al., 2020 (strawberry); Silwal et al., 2017 (apple)], pest and weed control [Raja et al., 2020 (tomato and lettuce); Oberti et al., 2016 (grape); Åstrand and Baerveldt, 2002 (sugar beet); Blasco et al., 2002 (lettuce)], spraying [Hejazipoor et al., 2021 (Anthurium); Gonzalez-de-Soto et al., 2016 (wheat); Adamides et al., 2017 (grape)], and pruning [Zahid et al., 2020 (apple); Chonnaparamutt et al., 2009; Ishigure et al., 2013 (cedar and hinko trees)]. Together with imaging and sensing, autonomous robotic systems are also deemed essential and integral parts for high-throughput plant phenotyping, as they will enhance substantially the capacity, speed, coverage, repeatability, and cost-effectiveness of plant trait measurements.

In this paper, we reviewed the latest development of robotic technologies in high-throughput plant phenotyping. We define the robotic technologies as a system having three components: (1) a sensing module that senses the target (plants or crops) and its environment, (2) a computational module to interpret the sensed information and form adaptive (or context-specific) decisions, and (3) an actuation module to complete certain desired operations (e.g., robotic probing, trait measurements, and navigation). For example, the robot makes decision based on the existing status of environment, obstacles, and plant geometry to manipulate a robotic arm to locate an imaging system with less occlusion and collision free close to plant organs, find appropriate target point on the leaf and control the end-effector based on the leaf angle for effective grasping, or accurately navigate the ground-based vehicles between crop rows. With this definition, systems like LemnaTec’s conveyor-based phenotyping platform (Fahlgren et al., 2015; Ge et al., 2016) was not considered in the review, because the plant movement usually follows a pre-defined schedule and no adaptive decision is made during phenotyping. Also not considered in this review are self-propelled ground vehicles or unmanned aerial vehicles (Bai et al., 2016; Han et al., 2018) that are merely used as a sensor deployment platform with no automated path planning or navigation.

Different artificial intelligence (AI) technologies such as deep learning, fuzzy logic, and genetic algorithms are actively used for control of the phenotyping robots. In recent years, deep learning techniques has gained increased interest to guide robotic manipulators and mobile platforms. In this regard, deep neural networks (DNNs) are commonly used to detect different objects in images such as crop rows, plant organs, soil, and obstacles. DNNs are typically operate directly on raw images and actively learn a variety of filter parameters during the training of a model (Pound et al., 2017; Irshat et al., 2018). Aguiar et al. (2020) presented a DNN models to detect the vine trunks as reliable features and landmarks to navigate a mobile robot in a vineyard. Parhar et al. (2018) used variation of Generative Adversarial Network (GAN) to detect the stalk of sorghum in the field and grasp it by a robotic manipulator.

There are three motivations behind writing this review paper. Firstly, robotic technologies in agriculture have seen rapid advancement recently with many emerging applications in plant phenotyping. A timely review of the literature is warranted to summarize the newest development in the field. Secondly, there is large and growing interest from the plant breeding and plant science communities in how these new technologies can be integrated into research and breeding programs to improve phenotyping throughput and capacity (Furbank and Tester, 2011; Fiorani and Schurr, 2013; Araus and Cairns, 2014). Thirdly, robotic phenotyping has advanced through cross-disciplinary collaborations between engineers and plant scientists. Outlining capabilities, goals and interests across these two very different disciplines may help readers to identify research gaps and challenges as well as provide insight into the future directions of the plant phenotyping robotic technologies.

## Review: Many Indoor and Outdoor Robots Were Developed to Measure a Wide Range of Plant Traits

Phenotyping robotic systems have emerged to automate the phenotyping process in different aspects. The robotic manipulators and ground-based vehicles are used as platforms to attach different sensors to collect data rapidly and with higher repeatability. Robotic systems are deployed to collect and measure the human-defined phenotypic traits (such as plant height, and leaf area). Additionally, in some cases it is needed to collect repeated measurements of plant traits within large populations at several time points during a growing season. Robotic systems are highly desirable in this scenario as they provide the necessary speed and accuracy for this kind of phenotyping tasks.

**FIGURE 1:**
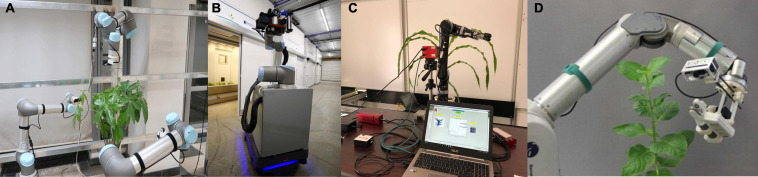
Plant phenotyping robotic systems for indoor environment: **(A)** A multi-robot system equipped with deep learning technique to determine optimal viewpoints for 3D model reconstruction (Wu et al., 2019), **(B)** Sensor-equipped robot to measure the reflectance spectra, temperature, and fluorescence of leaf (Bao et al., 2019c), **(C)** Robotic system to measure leaf reflectance and leaf temperate (Atefi et al., 2019), and **(D)** Robotic system for direct measurement of leaf chlorophyll concentrations (Alenyá et al., 2014).

Robotic platforms for plant phenotyping applications can be divided into two categories: those developed for indoor or controlled environments (greenhouse or laboratory), and those for outdoor environments (field) (Shafiekhani et al., 2017). In controlled environment, plants are either placed in a fixed position and the robot moves around the facility to interact with the plants, or the plants are moved by conveyor belts or other automated systems to a fixed location where the robot operates. Often the robotic system does not need to touch the plants. The robotic arm is equipped with RGB cameras or depth sensors [Time of Flight (TOF) cameras or 3D laser scanners] to acquire visible images or point cloud data. The morphological traits of the plants are then estimated from the reconstructed 3D model of the plants. Stem height and leaf length of corn seedlings were measured using a robotic arm at a fixed position and a TOF camera (Lu et al., 2017). Chaudhury et al. (2017) developed a gantry robot system consisted of a 3D laser scanner installed on the end-effector of a seven Degree of Freedom (DOF) robotic arm to compute the surface area and volume of *Arabidopsis* and barley. The settings of both robotic systems were unable to position the vision system to capture images from the leaves hidden by other leaves or the stem. This occlusion problem is common in image-based phenotyping (Das Choudhury et al., 2019). Even with imaging from multiple views (e.g., enabled by rotating plants during image acquisition), occlusion can still be substantial. The use of imaging systems carried by a robotic manipulator can provide viable solution to this issue, due to the flexibility of the robotic manipulator to position and orient cameras at the best intended viewpoints. Wu et al. (2019) proposed an automated multi-robot system, which comprised of three robotic arms each equipped with a depth camera to obtain the point cloud data of the plant (Figure 1A). Deep learning based next-best view (NBV) planning pipeline was presented to evaluate and select the next-best viewpoints to maximize the information gain from the plant in data acquisition process. The robotic arms then were manipulated based on the determined optimal viewpoints. Their system was more efficient and flexible compared to other robotic systems to address the occlusion issue. The ability of the system to find the optimal viewpoints, however, can be challenging, because its performance depends upon the predictions produced by the trained deep networks. This means that the best view-points may not be determined by the system if the deep networks can not generate accurate predictions.

**FIGURE 2:**
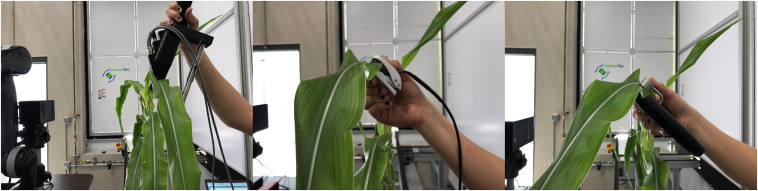
Manual measurements of leaf reflectance **(left)**, leaf temperature **(middle)**, and chlorophyll content **(right)** (Atefi et al., 2019).

A second group of indoor plant phenotyping robots sought to touch or probe plants or plant organs, in order to extend the ability of robotic phenotyping from plant’s outward morphological traits to innate physiological and biochemical traits (Schulz and Baranska, 2007; Biskup et al., 2009). In this sense, the phenotyping robot was designed to mimic humans to manipulate plants and measure certain traits from targeted plant organs (Figure 2). This type of the robotic systems usually included a robotic gripper designed to attach specialized plant sensors, and a vision module to segment the plant from the background and find an appropriate point on the organs for probing [Alenyà et al., 2011; Shah et al., 2016; Bao et al., 2017 (Ficus plant)] or grasping process [Alenya et al., 2013; Ahlin et al., 2016 (Anthurium, Pothos, and Dieffenbachia)]. A sensor-equipped robot was presented to measure physiological parameters of the plant (Bao et al., 2019c). The sensor unit including RGB, hyperspectral, thermal, and TOF cameras, and a fluorometer were attached to a robotic arm. The robot measured the reflectance spectra, temperature, and fluorescence by imaging the leaf or placing probes with millimeter distance from the leaf surface (Figure 1B). Two different plant phenotyping robotic systems were introduced to measure leaf and stem properties of maize and sorghum plants (Atefi et al., 2019, 2020). The systems consisted of a TOF camera, a four DOF robotic manipulator, and custom-designed grippers to integrate the sensors to the robotic manipulator. Image-processing and deep-learning based algorithms were presented to find the grasping point on leaves and stem. An optical fiber cable (attached to a spectrometer) and a thermistor were used to collect leaf hyperspectral reflectance and leaf temperature simultaneously. The stem diameter was measured by a linear potentiometer sensor. Leaf hyperspectral reflectance was used to build predictive models for leaf chlorophyll content, water content, N (nitrogen), P (phosphorus), and K (potassium) concentrations (Figure 1C). Alenyà Ribas et al. (2012) mounted a SPAD meter to a robotic arm to directly measure leaf chlorophyll concentrations of Anthurium White, Anthurium Red, and Pothus (Figure 1D). Quadratic surface models were applied to segment leaves from infrared-intensity images and depth maps captured by a TOF camera. The estimation issues of probing point caused by poor leaf-fitting model reduced the probing success rate of the robotic system (82%).

Although controlled environments can make it easier to grow plants and quantify their phenotypic traits, because environment plays a large role in determining plant traits, plants grown in controlled environments show many differences from plants grown in field conditions. Therefore, with the exception of a growing range of horticultural crops where production occurs in control environments, for many crops the assessment of phenotypic responses in field conditions provides more directly actionable information for crop improvement. A wide range of platforms have been developed for field-based high-throughput plant phenotyping [Montes et al., 2007; White et al., 2012; Gao et al., 2018 (soybean); Weiss and Biber, 2011 (detection and mapping of maize plants); Jenkins and Kantor, 2017 (stalk detection of sorghum); Iqbal et al., 2020 (plant volume and height); Smitt et al., 2020 (fruit counting of sweet pepper and tomato)]. These robotic systems are guided between crop rows and moved toward plants. This creates several new challenges for both navigation and data collection which are absent when robotic phenotyping is conducted in control conditions. Factors like temperature, sunlight, wind, and unevenness of soil surface, can negatively impact the performance of the system. Therefore, the hardware and software of the robotic system must be designed to be resilient to the unique challenges of operating in field conditions. In the field plants are always stationary, necessitating that (1) phenotyping robots move to the plants rather than vice versa, (2) all components of the phenotyping robot including the vision system, robotic arm, and sensors as well as power supplies be carried by a robotic mobile platform, and (3) this platform be capable of navigation whether through global positioning system (GPS) data and/or employing sensors to perceive its local environment to guide navigation.

**FIGURE 3:**
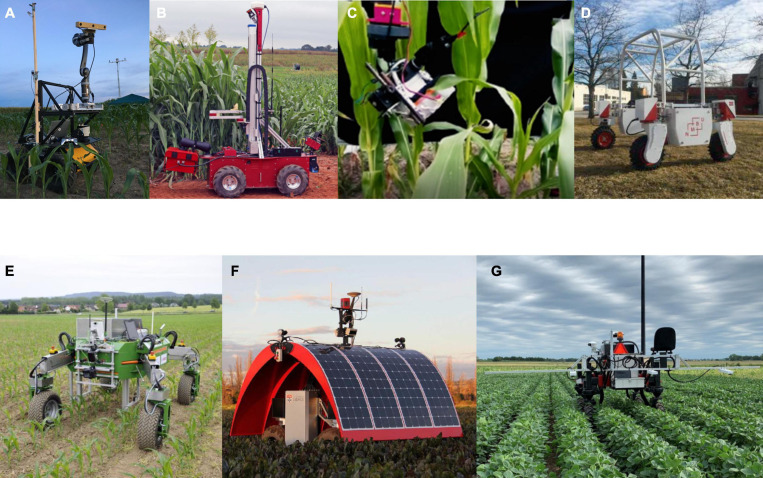
Plant phenotyping systems for outdoor environment: **(A)** Vinobot: robotic system including six DOF robotic manipulator and a 3D imaging sensor mounting on a mobile platform to measure plant height and LAI (Shafiekhani et al., 2017), **(B)** Robotanist: UGV-based robotic system equipped with a three DOF robotic manipulator and a force gauge for stalk strength measurement (Mueller-Sim et al., 2017), **(C)** A robotic system to slide LeafSpec across entire leaf to collect its hyperspectral images (Chen et al., 2021), **(D)** Thorvald II: VIS/NIR multispectral camera mounted on a mobile robot to measure NDVI (Grimstad and From, 2017), **(E)** BoniRob: autonomous robot platform using spectral imaging and 3D TOF cameras to measure plant height, stem thickness, biomass, and spectral reflection (Biber et al., 2012), **(F)** Ladybird: ground-based system consisted of a hyperspectral camera, a stereo camera, a thermal camera, and LIDAR to measure crop height, crop closure, and NDVI (Underwood et al., 2017), and **(G)** Flex-Ro: high-throughput plant phenotyping system equipped with a passive fiber optic, a RGB camera, an ultrasonic distance sensor, and an infrared radiometer for the measurement of NDVI, canopy coverage, and canopy height (Murman, 2019).

Unmanned ground vehicle (UGV) robotic systems employ a range of sensor types including light detection and ranging (LIDAR) and cameras [RGB, TOF, near infrared (NIR), and stereo vision] for data collection. They can be installed on a fixed stand within the overall mobile platform, or affixed a robotic arm to increase the number of diversity of positions from which sensor data can be collected. Different techniques such as 3D reconstruction, image processing, and machine learning are used for data analysis and quantify morphological traits. Existing UGV robotic systems have been employed to measure plant height, plant orientation, leaf angle, leaf area, leaf length, leaf and stem width, and stalk count of various species such as maize, and sorghum, sunflower, savoy cabbage, cauliflower, and Brussels sprout (Jay et al., 2015; Fernandez et al., 2017; Baweja et al., 2018; Choudhuri and Chowdhary, 2018; Vázquez-Arellano et al., 2018; Vijayarangan et al., 2018; Bao et al., 2019b; Breitzman et al., 2019; Qiu et al., 2019; Young et al., 2019; Zhang et al., 2020), count the cotton bolls (Xu et al., 2018), architectural traits and density of peanut canopy (Yuan et al., 2018), berry size and color of grape (Kicherer et al., 2015), and shape, volume, and yield estimation of vineyard (Lopes et al., 2016; Vidoni et al., 2017). A compact and autonomous TerraSentia rover equipped with three RGB cameras and a LIDAR was demonstrated to acquire in-field LIDAR scans of maize plants to extract their Latent Space Phenotypes (LSPs) (Gage et al., 2019). They were inferred from the images using machine learning methods (Ubbens et al., 2020) and contained information about plant architecture and biomass distribution. Shafiekhani et al. (2017) introduced a robotic system (Vinobot) including a six DOF robotic arm with a 3D imaging sensor mounted on a mobile platform. The Vinobot collected data in order to measure plant height and leaf area index (LAI) of maize and sorghum (Figure 3A). The authors reported that the use of a semi-autonomous approach created most of challenges for navigation of the system. In this approach, the alignment of the robot with the crop rows was required before autonomously moving between the rows and collecting data from the plants.

Measurements of some biochemical and physiological properties require a direct contact between sensors and plants. Measuring these properties therefore requires a robot capable of grasping or touching plant organs. Grasping plant organs in turn requires a dexterous robotic arm as well as onboard sensors and algorithms capable of reconstructing the 3D geometry of the target plant. Robotanist, a UGV equipped with a custom stereo camera, was established to measure stalk strength of sorghum (Mueller-Sim et al., 2017; Figure 3B). A three DOF robotic arm along with a special end-effector was mounted on Robotanist. The end-effector consisted of a rind penetrometer that was modified by attaching a force gauge and a needle. When the stalk was grasped by the end-effector, the needle and force gauge were pushed into the stalk to accomplish the measurement. The authors suggested to develop algorithms using laser scan and navigation camera data to improve the performance of the navigation system to reliably work under taller sorghum canopy and throughout the entire growing season. Abel (2018) attached a spectrometer to the robotic manipulator of Robotanist to capture spectral reflectance measurements of leaves and stems of sorghum. Random sample consensus (RANSAC) method was used for leaf and stem detection. A machine learning approach was applied to estimate the chlorophyll content of leaves, and moisture and starch contents of stems from reflectance spectra. Two factors reduced the grasping success rate of leaves (68%). First, the grasping process was failed because the wind moved the leaves and changed the position of the grasping point. Second, the occlusion and overlapping affected the performance of the segmentation algorithms to detect more leaves in the images. Chen et al. (2021) developed a robotic system including LeafSpec (invented at Purdue University) attached to a robotic manipulator to collect hyperspectral images of maize leaves in the field (Figure 3C). The robot slid the LeafSpec across the leaf from the beginning to tip to acquire hyperspectral images of entire leaf. The system predicted leaf nitrogen content with *R*^2^ = 0.73.

Other autonomous ground-based systems were presented to measure both morphological and biochemical/physiological attributes. A visible and near infrared (VIS/NIR) multispectral camera was mounted on a mobile robot called “Thorvald I” to measure the normalized difference vegetation index (NDVI) of wheat from multispectral images (Burud et al., 2017). The robot then modified to a new version called “Thorvald II” to have better performance for phenotyping tasks (Grimstad and From, 2017; Figure 3D). BoniRob was proposed as an autonomous robot platform including spectral imaging and 3D TOF cameras which can be used to measure plant parameters such as plant height, stem thickness, biomass, and spectral reflection (Ruckelshausen et al., 2009; Biber et al., 2012; Figure 3E). Underwood et al. (2017) introduced a ground-based system (Ladybird) for row phenotyping of grain and legume crops (wheat, faba bean, lentil, barley, chickpea, and field pea) (Figure 3F). Crop height, crop closure, and NDVI were determined after processing the data from the LIDAR and the hyperspectral camera. Flex-Ro, a multi-purpose field robotic platform was used for high-throughput plant phenotyping to measure phenotyping traits of soybean (Murman, 2019; Figure 3G). Three sets of sensors were installed on Flex-Ro to collect data from crop rows. For each set, a passive fiber optic cable, a RGB camera, an ultrasonic distance sensor, and an infrared radiometer were used to measure NDVI, canopy coverage, canopy temperature, and height.

Table 1 summarizes the indoor and outdoor robotic systems which could successfully measure plant traits for different crops.

**TABLE 1:** Summary of indoor and outdoor robotic systems that successfully measured plant properties for different crops.

Robot type	References	Species	Plant trait	Performance	Measurement method	Software system
Indoor	Alenyà Ribas et al., 2012	Anthurium Andreanum (White), Anthurium andreanum (Red), Epipremnum aureum (Pothos)	Leaf chlorophyll content	SR = 90%, 85%, 70%	Contact based	ROS
	Lu et al., 2017	Maize	Stem height, leaf length	ER = 13.7%, 13.1%	Non-contact based	Qt development environment (C++)
	Atefi et al., 2019	Maize, Sorghum	Leaf chlorophyll content, leaf potassium content, leaf water content, leaf temperature	*R*^2^ = 0.52, 0.52, 0.61 *R*^2^ = 0.58, 0.63	Contact based	MATLAB
	Atefi et al., 2020	Maize, Sorghum	Stem diameter	*R*^2^ = 0.98, 0.99	Contact based	MATLAB
Outdoor	Jay et al., 2015	Sunflower, Savoy cabbage, cauliflower, Brussels sprout	Plant height, leaf area	*R*^2^ = 0.99, 0.94	Non-contact based	Not reported
	Shafiekhani et al., 2017 (Vinobot)	Maize, sorghum	Plant height	*R*^2^ = 0.99	Non-contact based	ROS
	Abel, 2018 (Robotanist)	Sorghum	Stem starch content, stem moisture content, leaf chlorophyll content	*R* = 0.81, 0.72, 0.92	Contact based	ROS
	Baweja et al., 2018 (Robotanist)	Sorghum	Stalk count, stalk width	*R*^2^ = 0.88, MAE = 2.77 mm	Non-contact based	ROS
	Choudhuri and Chowdhary, 2018	Sorghum	Stem width	Accuracy = 98.2%	Non-contact based	Python
	Vázquez-Arellano et al., 2018	Maize	Plant height	AME = 8.7 mm *SD* = 35 mm	Non-contact based	Not reported
	Vijayarangan et al., 2018	Sorghum	Leaf area, leaf length, leaf width	Relative RMSE = 26.15%, 26.67%, 25.15%	Non-contact based	ROS
	Bao et al., 2019b	Maize	Plant height, leaf angle	*R*^2^ = 0.96, 0.83	Non-contact based	Not reported
	Qiu et al., 2019	Maize	Plant height	RMSE = 0.058 m	Non-contact based	ROS
	Young et al., 2019	Sorghum	Plant height, stem width	AE = 15%, 13%	Non-contact based	Not reported
	Zhang et al., 2020	Maize	Stand counting	*R* = 0.96 *SD* = 6.76%	Non-contact based	Not reported

*ER, error ratio; SR, success rate, MAE, mean absolute error; RMSE, root mean square error; AME, absolute mean error; AE, absolute error; SD, standard deviation; ROS, robot operating system.*

**FIGURE 4:**
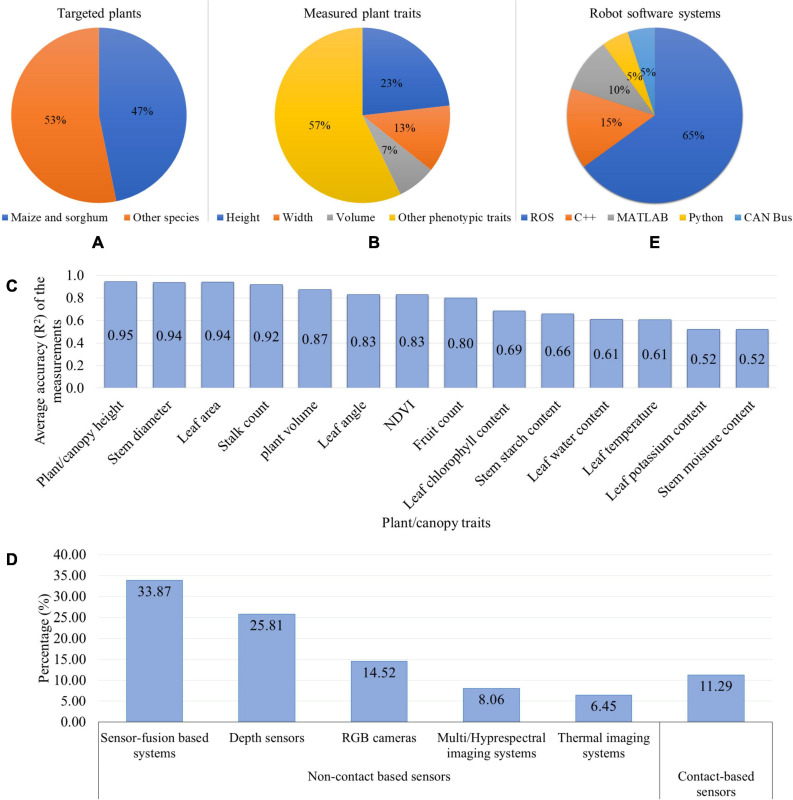
Summary statistics of the phenotyping robotic systems: **(A)** Targeted plants, **(B)** Plant/canopy traits measured by the robots, **(C)** Average accuracy (*R*^2^) to measure the phenotypic traits, **(D)** Robot vision/sensing systems, and **(E)** Robot software systems.

Figure 4 gives summary statistics regarding the plant phenotyping robotic systems that is discussed in this section. It can be seen that the robotic phenotyping research targeted maize and sorghum more than other species (soybean, wheat, barley, chickpea, pea, faba bean, lentil, cabbage, cauliflower, cotton, peanut, sunflower, grape, tomato, sweet pepper, and Arabidopsis) (Figure 4A). Maize and sorghum are two of the most economically important and highly diverse cereal crops with vast numbers of accessions (Zhao et al., 2016; Bao et al., 2019b). Therefore, more attention was devoted to breed Maize and sorghum to produce food, animal fodder, and biofuel. Moreover, the available genetic resources for these crops required the phenotyping data to map their genotypes to phenotypes and thus crop yield improvement. Accordingly, there has been an emerging need for phenotyping robots to automatically measure the phenotypic traits. Regarding the plant structure, maize, and sorghum have similar morphology. Their leaves are arranged alternately on each side of the stem that has cylindrical/elliptic-cylinder shape and is positioned in the middle part of the plant. This plant structure provides less complexity for the robotic system to distinguish between the stem and leaves and extract their features. Figure 4B shows that the height, width, and volume of plant/canopy are three main (morphological) traits that more frequently measured by the robotic systems than other traits, each of them being ≤ 5% (leaf length, leaf width, leaf angle, leaf area, leaf reflectance, leaf chlorophyll content, leaf/canopy temperature, LAI, plant/canopy NDVI, stem reflectance, stalk strength, stalk count, berry size, and fruit count). Two reasons can be considered for the frequent measurements of these phenotypic traits. Firstly, the plant architectural traits (such as plant height) are the most common and important parameters for field plant phenotyping since they have significant effects on light interception for photosynthesis, nitrogen availability, and yield (Barbieri et al., 2000; Andrade et al., 2002; Tsubo and Walker, 2002). Consequently, by studying and then manipulation of the plant architecture, the crop productivity will be increased. Secondly, as it was discussed in this section, the robot just needs non-contact based sensors (RGB camera or depth sensor) to collect data from the plants. Then, by analyzing the 2D images or creating plant 3D models, the aforementioned plant traits can be estimated in either ways: (1) the correlation between the pixel counts in the images and the ground truth measurements, or (2) extracting the distance/volume in real world from the depth sensor data. Hence, the measurement of these morphological properties is less challenging for the phenotyping robots using simple sensors and algorithms. In addition to the more frequent measurements of stem height and width (of maize and sorghum), these properties were also measured more accurately by the robotic systems because they are less affected by the plant morphology (Figure 4C). The first step to extract the stem height and width is to detect the stem and segment it from other plant organs. The morphology of maize and sorghum (alternately arranged leaves, and cylindrical-shaped stem in the middle) provides more hints for stem detection and segmentation. Moreover, the height and width can be measured as linear measurements. Accordingly, these two stem properties can be measured with less complexity and higher accuracy. Figure 4D illustrates that non-contact based sensing systems (such as RGB, stereo vision, and multispectral cameras, and LIDAR) were used more in phenotyping robots compared to contact-based sensors (chlorophyll meters, spectrometers, thermistors, and linear potentiometers). This can be explained by the fact that the majority of the robotic systems were developed to measure the morphological traits or some physiological properties. To achieve these goals, the phenotyping robots are required to use the 2D images/3D models of plants using non-contact based sensors. Among the non-contact sensors, the sensor-fusion based systems (including RGBD/stereo vision cameras, RGB camera + LIDAR, RGB + TOF cameras, spectral imaging + TOF camera) and depth sensors (TOF camera, LIDAR, laser scanner, and ultrasonic sensor) were commonly used as vision/sensing systems for the phenotyping robots. The key is to acquire depth information as a vital parameter to manipulate a robotic arm to grasp the plant organs, navigate a mobile robot between crop rows, and measure plant properties (such as height, width, and volume). Sensor-fusion based systems were employed by phenotyping robots more often than depth sensors. The reason would be that these sensors prepare the plant color/spectral information along with the depth information. Consequently, by acquiring more information, the plant can be effectively segmented from the background and the plant properties can be effectively measured. Regarding the robot software system, it can be found that Robot Operating System (ROS) is one the most popular systems to develop the software of the phenotyping robots (Figure 4E). ROS is an open source system that provides services, libraries, and tools for sensors-actuators interface, software components communication, and navigation and path planning^1^. Different manufacturers of robot’s hardware provide ROS drivers for their products such as imagery systems, sensors, actuators, robotic manipulators, and mobile platforms. This allows the researchers to develop the phenotyping robotic systems more efficiently.

Regarding the platform of the mobile phenotyping robots, most of the mobile platforms were developed by researchers (86%) and few off-the-shelf robots were used (14%). The custom-designed platform offers potential to meet specific conditions regarding soil, weather, and plant which vary with experimental site and phenotyping task. Moreover, the researcher has more control on modifying the hardware and software. Based on the reviewed papers, most of the mobile platforms used four wheels/legged-wheels (88%) as their driving system compared with tracked mechanism (12%). In the wheeled-vehicles, the wheels can be independently steered (good maneuverability) which provides high flexibility with respect to control and navigation (desired orientation angle and rotation speed) of the vehicles between the crop rows in the field. Moreover, the vehicle can move faster using (legged) wheels and has high ground adaptability (crop height, and irregular and sloped terrain) using legged-wheels. However, the tracked-vehicles create more traction and less pressure on soil (work better in wet soil and less soil compaction), and can drive over rough terrain and obstacles easier than the wheeled-vehicles (Bruzzone and Quaglia, 2012). Accordingly, a hybrid locomotion system can be developed with the combination of legged-wheels and tracked systems. Therefore, this platform can use the advantages of the both driving systems to accomplish phenotyping tasks more effectively and efficiently.

## Phenotyping Robots Face Several Challenges

There are several outstanding challenges in the development of robotic systems for plant phenotyping. Some of these challenges related to segmentation (vision systems) and grasping (robotic manipulators) are shared or at least similar for both indoor and outdoor phenotyping robots. Other challenges, particularly those related to navigation are specific to outdoor robotic phenotypic applications.

### Complex and Deformable Nature of Plants Represents a Major Issue for Robot’s Vision and Sensing System

The UGV or robotic manipulator equipped with contact/non-contact based sensing systems offer a great potential to measure plant phenotypic data compare to non-autonomous robotic sensing systems. For example, the UGV equipped with stereo vision camera can move between crop rows and collect images from the canopy or individual plant. The image data can be analyzed immediately or can be processed later to extract plant properties. The long-term measurement of the plant traits can provide useful knowledge for crop modeling purposes over time (Duckett et al., 2018). Another example would be the robotic manipulator equipped with a hyperspectral imaging system. The robotic arm can move around the plant to locate the sensor close to the plant organs. With this proximal sensing, more phenotyping information can be acquired about the organs. However, the robotic vision/sensing technologies for the phenotyping task encounter different challenges.

Various imaging technologies are utilized as vision systems of the robots. Visible imaging/RGBD camera are commonly used technologies that rely on the color/texture information of an object. Images are processed to segment plant organs and identify desirable targets for grasping. The identification and localization of different plant organs (such as leaves, stems, flowers, and fruits) is one of the major problems in computer vision, due to complex structure and deformable nature of plants. The overlap between the adjacent leaves or leaf-stem causes occlusion; even though leaf and stem have different architecture, they share similarities in color and texture. Accordingly, it is difficult to distinguish occluded leaves or stem in the image. The morphology of plants (shape and size) varies dramatically across different plant species and even within a single species different varieties or the same variety grown in different conditions may exhibit radically different morphology. In this regard, the software of the robotic system should cover a wide range of scenarios and possibilities to be able to respond and adapt appropriately to day-to-day changes in the same plant or differences between plants within the same experiment. Additionally, non-uniform imaging conditions (lighting and background) make it more complex to find an appropriate color space and optimal approach for the segmentation purposes (Zhang et al., 2016; Narvaez et al., 2017; Qiu et al., 2018; Bao et al., 2019a).

Multispectral/hyperspectral and thermal imaging systems are sensitive to illumination since the reflectance from the plant organ is depend on its distance and orientation toward the light source/incident radiation and camera. Moreover, multiple reflectance and also shade will occur due to the curvature nature and complex geometry of plant (Li et al., 2014; Mishra et al., 2017; Qiu et al., 2018). To deal with these issues, researchers introduced different technical solutions. Behmann et al. (2016) combined the hyperspectral image with 3D point cloud (using a laser scanner) of sugar beet to create hyperspectral 3D model. Then, it was used to quantify and model the effects of plant geometry and sensor configuration. Finally, the geometry effects in hyperspectral images were weakened or removed using reflectance models. Shahrimie et al. (2016) used inverse square law and Lambert’s cosine law along with Standard Normal Variate (SNV) for maize plants to remove the distance and orientation effects.

### Robotic Control System Needs to Deal With Dynamic and Unstructured Environment

The size and orientation of the plant organs are constantly changing across their growth stages. Therefore, the lack of needed DOF or enough workspace of the robotic manipulator are the limitations for the robots to grasp the plant organs and sense their properties successfully. The robotic arm cannot reach the organs if they are out of its workspace. In addition, a robot arm with less flexibility (DOF) might not able to properly adjust the angle of its end-effector in grasping process.

Field-based robots need to navigate between crop rows and then turned to the next row safely and autonomously. To achieve this task, the crop rows and obstacles should be detected to build a map of the surrounding area. Then, their position and orientation relative to the vehicle will be found to compute an optimal path and avoid unexpected obstacles. Finally, adequate action will be determined to steer the wheels and guide the system around the field. However, the uncontrolled and unstructured field environment creates challenges for accurate navigation and control of the robot (De Baerdemaeker et al., 2001; Nof, 2009; Bechar and Vigneault, 2016; Shamshiri et al., 2018). GPS is a common method for robot navigation. However, the tall plant canopy affects on the accuracy of GPS for navigation purposes as the canopy blocks the satellite signals to the GPS receiver. Hence, the information provided from other sensors along with GPS is also required to detect the obstacles, precisely guide the phenotyping robot, and minimize the damage to the robot and plants. The UGV based phenotyping robots can facilitate the data fusion of GPS and other sensors since the robot can equipped with various sensors (such as LIDAR, RGB/stereo vision cameras) and also precisely control their location (Duckett et al., 2018). Nonetheless, varying ambient light conditions, changing crop growth stages (size, shape, and color), and similar appearance between crops and weeds are common factors that fail visual navigation. In these situations, RGB sensor-based systems usually cannot find a stable color-space or plant features to detect different objects. Incomplete rows and missing plants can cause errors to compute distance between the robot and plants using range sensors. Different soil properties (soil types and moisture) and terrain variation (even, uneven, flat, slope) are other factors that influence robot dexterous manipulation, wheel-terrain interaction, wheel slip, and steering control algorithms (Åstrand and Baerveldt, 2005; Grift et al., 2008; Li et al., 2009; Shalal et al., 2013).

After navigating the robot between the rows, a suitable path should be selected for the robotic manipulator with minimum collisions inside a plant or canopy to reach and grasp the targets delicately. However, robots operate in extremely complex, dynamic, uncertain, and heterogenous real world condition. In this situation, visual occlusion of a plant by others caused by high plant density should be taken into account for target identification and segmentation. In addition, the target information will be affected by sunlight and wind. For instance, TOF/RGBD cameras use infrared light to measure distance. Since the sunlight has infrared wavelengths and wind moves the targets, the location of the target in 3-dimensional space might not be accurately measured (Andújar et al., 2017; Narvaez et al., 2017; Qiu et al., 2018; Li et al., 2020a). Consequently, the obstacle-avoidance path-planning algorithm cannot be determined correctly. Another example would be when the targets are seen shinier or darker because of specular reflection or shade.

### Issues With Robot Software for Phenotyping Robotic System Development

Two main drawbacks present in many robot software are: (1) the lack of support for certain functional packages (of open source software) and (2) real-time constraints (Barth et al., 2014; Park et al., 2020). For the first issue, it can be supposed that a phenotyping robot is developed by researchers to accomplish a phenotyping task. They create the robot library and share their codes with the (open source) software community. However, by ending the project, there is no guarantee to fix the bugs and update the codes. In the case of other researchers might start similar research using the shared codes, it might be problematic to make the research forward because of the lack of support for the robot library. The second challenge is the real-time constraints that causes system malfunction due to latency. One example would be when a UGV moves between crop rows to measure plant traits. If the robot cannot satisfy the real-time constraints, the robot will have delay to identify the obstacles or adjust its position relative to the crop rows. Accordingly, the robot could hit the obstacles and the plants and this causes the physical damage to the robot or plants. Regarding ROS, although ROS1 has real-time constraints, however the community is actively working on software improvement. For example, RT-ROS supports the real-time communication that leads to performance enhancement of ROS1 (Wei et al., 2016). It is obvious that by growing the ROS community, sophisticated libraries and packages will be developed for more plant phenotyping applications.

### Other Challenges: Managing Big Data, Reliable Power Source, Durability Under Harsh Environment, and High Cost

The phenotyping robot collects massive volumes and various types of data (such as images, multi/hyperspectral data) taken by different sensors from large population of plants. The robot needs to analyze large quantities of data in real-time for suitable action/decision-making process. In addition, the large-scale phenotypic data could be stored properly for the benefit of future research. Therefore, managing and analyzing the big data as a result of high-throughput, robotically collected plant traits is an emerging issue for the phenotyping robot.

The field-based mobile robots need to be equipped with reliable power sources to provide energy for the vehicle carriage weight, distance traveled, and different electrical components such as sensors for data collection. Batteries are commonly used for this purpose. The problems with batteries are: (1) limited operating time that prevents the robots to work for long time and accomplish large-scale missions, and (2) need to recharge which typically takes a long time.

Another challenge is the durability and stability of these robotic systems under harsh outside environment caused by extreme temperature, high humidity, strong sunlight, and dust. These harsh conditions can cause damages for the components of the robotic system and accordingly will have negative effects on the robot’s performance.

The cost of phenotyping robots (in general agricultural robots) is still high and this makes limitations for wide-spread use of the robots. In most cases the phenotyping robotic systems are developed for research purposes and the robots are not commercially available yet. Both the hardware and software systems were restricted to a very specific condition and could not be transferred to a different scenario. This leads to high R&D (research and development) cost that can not be spread over multiple units. However, more general purpose phenotyping robots can be developed and commercialized in the future and their cost will be reduced substantially. Moreover, with the consistent trend of price reduction of electronics, sensors, and computers, the robotic systems will become cost-effective enough to be more widely used for phenotyping tasks.

## Potential Improvements of Phenotyping Robots

### Sensors and Controllers Fusion Technique Can Improve the Performance of Robot

Sensing-reasoning, and task planning-execution are two essential functions for autonomous phenotyping robots. They sense the environment, apply an appropriate control algorithms, make decision, and act in real-time to perform the phenotyping tasks (Grift et al., 2008; Bechar and Vigneault, 2016). The design of the phenotyping robot and its control algorithm needs to be optimized to achieve successful operation in continuously changing environment. To reach this purpose, the phenotyping robots need to employ advanced technology to cope with the dynamic and unstructured environment. The emerging sensor technologies such as sensor fusion increase the robot capabilities and yield better results (Grift et al., 2008). Sensor fusion allows the robot to combine information from a variety of sensing modules to form better decision for navigation and path planning, as well as increase the capacity of sensing to gather more information from the plants. For example, Choudhuri and Chowdhary (2018) measured the stem width of sorghum with 92.5% accuracy using RGB data. However, they achieved higher accuracy (98.2%) after combining RGB + LIDAR data. Kim et al. (2012) could successfully navigate an unmanned weeding robot using sensor fusion of a laser range finder (LRF) and an inertial measurement unit (IMU). The robot also needs more sophisticated and intelligent algorithms to accomplish different subtasks such as sensing, navigation, path-planning, and control. Different control strategies such as genetic algorithm (GA), fuzzy logic (FL), neural network (NN), reinforcement learning (RL), and transfer learning (TL) can be integrated to develop such robot algorithms (Shalal et al., 2013). Therefore, a robust controller will be provided for the phenotyping robot since the robot control system can use the merits of both technologies (combining two control strategies). Batti et al. (2020) studied the performance of fuzzy logic and neuro-fuzzy (NN + FL) approaches to guide a mobile robot moving between the static obstacles. The authors found that neuro-fuzzy controller provide better results for robot navigation compare to fuzzy logic controller. Although several different autonomous phenotyping robots were developed, more research is needed to adapt and improve the advanced technologies to overcome the robot limitations to accomplish the phenotyping tasks, and also increase the autonomy level of the phenotyping robots.

### Internet of Robotic Things (IoRT): Technology to Manage Big Data for Phenotyping Robots

Internet of Things (IoT) technologies are helpful to send lots of data collected by different sensors over Internet in a real-time manner. The Internet-of-Robotic-Things (IoRT) is the confluence of autonomous robotic systems with IoT which is an emerging paradigm that can be employed for phenotyping robots (Grieco et al., 2014; Ray, 2016; Batth et al., 2018; Saravanan et al., 2018; Afanasyev et al., 2019). Mobile robots can use IoT to transfer and store a large amount of phenotypic datasets to a central server. By sending the data via IoT, the robots do not need to frequently move to a place and physically upload the collected data to a local server/computer. Moreover, plant breeders/scientists can visualize the data using a mobile device (a tablet or a smartphone) or an office computer and therefore the performance of plants and changes in crop growth and development can be remotely inspected in different regions of the field in a real-time fashion. Another attractive aspect of using IoRT is to send commands to robots to accomplish phenotyping tasks. For instance, an operator can remotely control the greenhouse robotic manipulator systems via Internet any time from his home/office to collect phenotypic data. Another example is when the close inspection of an area in a field is necessary after analyzing the drone-based image data; therefore, commands can be sent via Internet to deploy mobile robots in this regard. Several mobile robots can work together to operate more efficiently to achieve a specific task.

### Solar Panels and Hydrogen Fuel Cell: Renewable Power Sources for Phenotyping Robots

Solar panels and hydrogen fuel cell are two technologies that produce clean, renewable, and sustainable energy. A solar panel consists of many small units called photovoltaic cells which convert sunlight into electricity. The maintenance cost of the solar panel is low since it does not have moving parts (no wear) and it just need to clean the cells. The hydrogen fuel cell comprised a pressurized container to store hydrogen. The fuel cell is an electrochemical device that takes oxygen from the air and combines hydrogen with oxygen to produce electricity. Re-fueling time of a hydrogen fuel cell is very short (5 min or less) and its cells are fairly durable.

Based on the advantages of solar panels and hydrogen fuel cell, both technologies can be used as renewable power sources for different components of the phenotyping robots (Underwood et al., 2017; Quaglia et al., 2020). However, there is not a wide range of application of these technologies for the phenotyping robots. The cost of both technologies is high. For solar panels, the efficiency of the system drops in cloudy and rainy days. In addition, more solar panels are needed to produce more electricity which requires a lot of space. For hydrogen fuel cell, there are relatively few places to re-fuel the cell. Nevertheless, both technologies are constantly developing which can be assumed to reduce their cost and improve their efficiency to produce electricity.

## Perspective Applications of Robotic Phenotyping

### Phenotyping Robots Has Great Potential to Measure Other Plant Properties

Section “Review: Many Indoor and Outdoor Robots Were Developed to Measure a Wide Range of Plant Traits” introduced the robotic systems for indoor and outdoor applications to measure several different plant traits. However, other leaf/stem characteristics are also reliable indicators to detect the symptoms of biotic/abiotic stresses and monitor the plant health during a growing season. Stomatal conductance, gas exchange, and chlorophyll fluorescence of leaves are indicative of their water status, photosynthesis, and chlorophyll content (Castrillo et al., 2001; Ohashi et al., 2006; Li et al., 2020b). Stem sap flow and lodging resistance can provide useful information about plant water use and stem strength (Cohen et al., 1990; Kong et al., 2013). These aforementioned phenotypic traits are still measured manually. On the other hand, new clip-on sensor system can be presented to measure them automatically. The system includes a custom-designed gripper/clip combined with novel sensor(s) (Afzal et al., 2017; Palazzari et al., 2017). The design of these sensing systems is important since the accuracy and robustness of trait prediction models depend on the phenotypic data quality (Würschum, 2019). The design of the gripper and DOF of the robotic manipulator should allow a good and gentle contact between the sensing unit and the leaf/stem. Sometimes a vacuum mechanism attached to a soft gripper can hold the leaf/stem and help the sensing unit for effective contact and collect accurate data with less damage to the plant organs (Hayashi et al., 2010; Hughes et al., 2016; Zhang et al., 2020). Moreover, autonomous robots should gather data with minimum error (high signal to noise ratio). Therefore, sensors with high signal to noise ratio should be selected and accurately calibrated. In addition to the accuracy, the robots should rapidly (short execution time) accomplish their missions. Deep reinforcement learning (DRL) technique is an accurate and reliable method to find an optimal path with nearest and collision avoidance route. This technique can be adopted by phenotyping robots to manipulate a robotic arm for grasping process or to navigate a mobile robot between crop rows (Zhang et al., 2015; Zhang et al., 2019; Duguleana and Mogan, 2016; Franceschetti et al., 2018; Taghavifar et al., 2019). Although the robotic phenotyping is mainly focusing on leaf and stem, it can be utilized for other plant organs such as inflorescences (spike, panicle, and tassel), flowers, fruits, and roots.

The morphometric parameters of inflorescence are highly correlated with yield and grain quality (Leilah and Al-Khateeb, 2005; Gegas et al., 2010). Several studies discussed about using image-based techniques (2D images/3D reconstruction) to extract architectural traits such as length and width of inflorescence, inflorescence volume (weight), grain shape and size, grain angle, and number of grains, and number of flowers (Faroq et al., 2013; Crowell et al., 2014; Gage et al., 2017; Rudolph et al., 2019; Sandhu et al., 2019; Xiong et al., 2019; Zhou et al., 2019). In such applications to measure the morphological traits, a robot with LIDAR/camera can be useful to automatically take images/point cloud data from different views of the inflorescence. The physiological traits are indicator for stress or disease. For instance, the temperature of the spikes was used for detecting the plant under the water stress (Panozzo et al., 1999). Conceivably, a robotic arm equipped with a temperature sensor can grasp the spike and insert the sensor into spikelets to record their temperature.

Several properties of fruits such as water content, sugar content, chlorophyll, carotenoid, soluble solid, acidity, and firmness are measured for fruit quality assessment. The spectroscopy/spectral imagery are non-destructive and high-throughput methods to estimate these qualitative parameters (Berardo et al., 2004; ElMasry et al., 2007; Shao and He, 2008; Wu et al., 2008; Nishizawa et al., 2009; Penchaiya et al., 2009; Ecarnot et al., 2013; Guo et al., 2013; Dykes et al., 2014; Wang et al., 2015; Mancini et al., 2020). However, a robotic system can be presented to monitor the dynamics of these attributes for hundreds of growing fruits per day. For example, a portable spectrometer can be attached to the robot’s end-effector. After detecting the fruit on the plant, the robot can grasp the fruit and gather its spectral data to further infer its quality parameters.

Since the root has functional roles in resource acquisition, the characteristics of root provide valuable information about plant physiological and ecosystem functioning (Mishra et al., 2016). In traditional root phenotyping, two different methods are used to acquire images from root (in the soil or soil-free or transparent media). In first method, a camera is mounted on a tripod and moved by a human around the root, and in the second method camera(s)/sensor(s) are set in fixed point(s) and root (plant) is rotated (Atkinson et al., 2019). This is a tedious task and some root information (such as fine branches) might be lost due to less flexibility of the system to take up close images from the complex architecture of root. Consequently, automated root phenotyping systems can facilitate and improve the traditional root phenotyping in terms of efficiency and effectiveness with acquiring fast and precise measurements (Wu et al., 2019). Here, the “plant to sensor” system can be used to examine vast number of roots (or plants) without the need of huge space of greenhouse facility. In this system, the root (or plant) is moved toward a robotic manipulator (equipped with camera/sensor) and located on a rotation table. In each step angle of the table, the root is rotated and stopped in front of the robotic system. Then, the robotic manipulator moves the camera around the root and gather close proximity data from different views (positions and angles). Therefore, more detailed information of root can be captured due to high resolution sensing offered by the robotic system.

### Robots in Greenhouses Complement the Image-Based Phenotyping

Automatic greenhouses such as LemnaTec (LemnaTec GmbH, Aachen, Germany) monitor plants using image-based technique. While it has shown great potential to measure and predict the plant traits, many hurdles cannot be handled by this technology. It needs to efficiently manage “big data” problems and also postprocess images to characterize the plant traits. Moreover, this approach is not sufficient for early detection of stress/disease with internal symptoms. Furthermore, this method requires direct measurements using sensors to calibrate and validate of its models to extract the phenotypic traits from images (Madden, 2012; Mutka and Bart, 2015; Singh et al., 2016; Lee et al., 2018). Hence, several robotic arms with different sensors can be integrated to the greenhouse for real-time and direct measurement of the chemical/physiological traits. Basically, plants are transported by an automatic conveyor belt and stopped in front of each robotic system. Then, the system uses “sensor-to-plant” concept (Lee et al., 2018) in which the robot moves toward the plant to take measurements before sending it through the imaging chambers. These stationary robotic systems are designed to operate in indoor environment. Moreover, several robots can be presented to collect data from a specific plant. It is difficult to develop a general prototype that are broadly applicable for different conditions (Mutka and Bart, 2015; Wu et al., 2019). However, the software and hardware of the robots should be adapted to other species and field-phenotyping applications. The challenge for both type of robots (indoor/outdoor) would be continuously collect and save large amount of data.

### Swarm Robot Is a New Frontier to Efficiently Accomplish Complex Phenotyping Tasks

**FIGURE 5:**
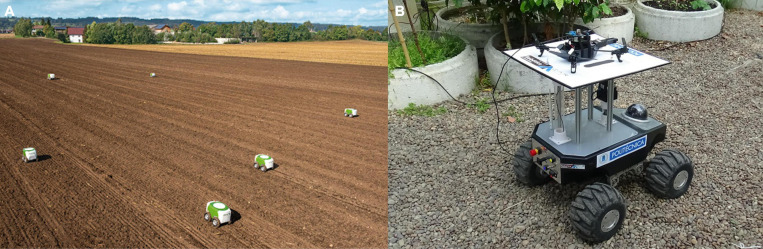
**(A)** Mobile Agricultural Robot Swarms (MARS) for seeding process (The European Coordination Mobile Agricultural Robot Swarms (MARS). PDF file. November 11, 2016. http://echord.eu/public/wp-content/uploads/2018/01/Final-Report-MARS.pdf), **(B)** UAV-UGV cooperative system to measure environmental variables in greenhouse (Roldán et al., 2016).

Swarm robotics is a new frontier technology which has potential application for proximal sensing of plants, and data/sample collection in a large field. A swarm robotics system composed of large numbers of autonomous robots that are coordinated with local sensing and communication, and a decentralized control system (Brambilla et al., 2013; Bayındır, 2016; Blender et al., 2016; Chamanbaz et al., 2017; Figure 5A). The application of swarm robots has some advantages which is suitable for large scale tasks. Since swarm robotics has large population size, the tasks can be decomposed using parallelism and can be completed efficiently and consequently it would save time significantly. Moreover, the swarm robots can achieve the distributed sensing that means they can have a wide range of sensing in different places at the same time (Navarro and Matía, 2012; Tan and Zheng, 2013).

Both UAV and UGV by itself have been successfully employed in plant phenotyping tasks. The coordination between UAV and UGV enables a new breakthrough application of UAV/UGV cooperative systems to achieve a common goal more effectively and efficiently (Arbanas et al., 2018; Vu et al., 2018). Both vehicles in this cooperative team share complementarities according to their capabilities that allow them to operate in the same field and work together to fulfill phenotyping missions. In this manner, the UAV can fly to quickly obtain overview of the fields beyond the obstacles; whereas the UGV can continuously patrols in the field with large payload capabilities of different sensors and robotic arms (Chen et al., 2016; Roldán et al., 2016; Figure 5B). In the context of UAV-UGV cooperation, an obstacle map of the field will be provided by the UAV for UGV path planning. Based on their communication and the map, the UGV can move rapidly between the crop rows for up-close plant investigation.

## Concluding Remarks

Autonomous robotic technologies have the potential to substantially increase the speed, capacity, repeatability, and accuracy of data collection in plant phenotyping tasks. Many robotic systems are successfully developed and deployed in both greenhouse and field environments, tested on a variety of plant species (row crops, specialty crops, and vineyards), and capable of measuring many traits related to morphology, structure, development, and physiology. Many technical challenges remain to be addressed regarding sensing, localization, path planning, object detection, and obstacle avoidance. Intensive research is needed to overcome these limitations of phenotyping robots and improve their speed, accuracy, safety, and reliability. Collaborations among different disciplines (such as plant science, agricultural engineering, mechanical and electrical engineering, and computer science) are imperative. With this transdisciplinary research, more efficient and robust sensing and control systems will be developed for intelligent plant phenotyping robots. Sophisticated sensor modules can be developed using sensor-fusion techniques. Regarding the control systems, multiple intelligent algorithms (such as different AI algorithms) can be combined to design more powerful controllers. These developments can potentially overcome the issues caused by changing environmental parameters, and complex structure of plants. Moreover, the suitable sensing and control systems yield better performance for accurate object detection (mainly for plants and crops, but also for humans, animals and other obstacles coexisting in the environments), path planning, and navigation. Sufficient funding from the public and private sources is the key to fuel the high-risk research in intelligent phenotyping robots in a sustainable way. We are optimistic that, in the next 10 years, we will see great leaps forward in autonomous and robotic technologies in plant phenotyping, enabled by the confluence of the rapid advancements in sensing, controllers, and intelligent algorithms (AIs).

## Author Contributions

AA and YG provided the conceptualization of the manuscript. AA drafted the manuscript. YG, SP, and JS substantially edited the manuscript. All the authors contributed to the article and approved the submitted version.

## Conflict of Interest

The authors declare that the research was conducted in the absence of any commercial or financial relationships that could be construed as a potential conflict of interest.

## Footnotes

1 https://www.ros.org/
